# Between political ambitions and professional practice

**DOI:** 10.1177/17449871261419023

**Published:** 2026-05-05

**Authors:** Sofia Nilsson

**Affiliations:** Registered Nurse, HKR, Sweden

## A recurring in-between space

In discussions about health and social care, the question frequently arises as to why political ambitions and professional practice do not always fully align. The goals are often shared, and the commitment to providing high-quality and equitable care is strong. Yet tensions emerge in the process of implementation, sometimes expressed as frustration, feelings of inadequacy, and a sense that different parts of the system are moving at different speeds.

With experience both as a registered nurse and as a political decision-maker at municipal, regional, and national levels, I have long found myself inhabiting this in-between space. It is a space where strategic direction meets everyday practice, where professional judgement encounters governance and policy signals, and where person-centred ambitions are sometimes tested against organisational and administrative demands. This text is an attempt to reflect on this field of tension, rather than to offer ready-made solutions.

## Two perspectives that depend on one another

Politics and professional practice operate on the basis of different mandates and responsibilities, which also shape their respective perspectives. Politics is tasked with formulating goals, prioritising resources, and ensuring accountability to citizens. Professional practice, in encounters with people, is responsible for translating these goals into action through knowledge, experience, and ethical judgement.

At their core, these are complementary roles. Difficulties arise when the boundary between them becomes blurred. When politics not only sets direction but also specifies in detail how work should be carried out, or when professionals experience that policy goals are insufficiently grounded in the realities of everyday practice, collaboration risks being undermined. The Swedish Trust Delegation, a government-appointed commission tasked with promoting trust-based governance and management in the public sector, has highlighted that detailed governance and extensive follow-up requirements risk constraining professionals’ scope for action and, consequently, weakening the ability to draw on professional knowledge and experience when addressing complex situations (Trust Delegation, 2018). In such cases, governance may be experienced as control, and professional discretion as restricted.

This challenge is not unique to health and social care, but its consequences become particularly evident in settings where relationships, continuity, and trust are fundamental to quality.

## Person-centredness as more than a way of working

Person-centred care has often been described as an approach grounded in the direct encounter between patient and professional. The focus is placed on the patient’s narrative, participation, and partnership. At the same time, both experience and research show that these ideals are difficult to fully realise if organisational and governance conditions do not support them (McCance and McCormack, 2025).

This raises the question of whether person-centredness can also be understood as a perspective on how systems are designed and led. If partnership is central to the care encounter, there may be reason to reflect on the relationship between decision-makers and services in similar terms. What forms of governance create conditions for responsibility, learning, and professional judgement, and which risk generating distance and mistrust?

## When governance becomes an obstacle rather than a support

In complex welfare systems, there is a tendency to respond to insufficient goal attainment with additional steering signals, more detailed requirements, and closer monitoring. The intention is often to strengthen quality, equity, and transparency. However, experience from many organisations suggests that this development can also have unintended consequences. The Swedish Agency for Public Management (2023) describes how this type of governance can lead to ambiguity regarding what is actually prioritised, particularly in systems where the state governs through a combination of legislation, financial incentives, and targeted assignments.

When the number of goals, assignments, and indicators is gradually expanded without previous requirements being phased out or prioritised, what is sometimes described as *goal congestion* emerges. For example, an organisation may have so many parallel goals that it becomes difficult for staff to determine which are most important to focus on. This can result in reduced effectiveness and lack of clarity about what should genuinely be prioritised.

Organisations are expected to meet a large number of parallel goals, formulated in different steering documents and with varying time horizons. Each goal is reasonable in itself, but together they risk competing for time, attention and resources.

In everyday practice, this may mean that professionals are forced to navigate between demands for accessibility, quality, efficiency, financial control, and documentation, whereas the space for reflection and adaptation diminishes. The focus risks shifting from what is most important for the patient and the service, to what is most readily measurable at a given moment.

For person-centred care, this is particularly challenging. Relationships, continuity, and shared goal-setting with the individual are core components, yet these are not always easily captured through standardised measures. When governance strongly rewards what is measurable, there is a risk that what is meaningful for the patient and professionally relevant in the encounter is pushed into the background.

## Goal congestion, prioritisation, and ethical judgement

The issue of goal congestion also carries an ethical dimension. Healthcare is a field in which prioritisation is continuously required, both at system level and in individual encounters. The ethical platform for priority setting (Government of Sweden, 1996) not only provides guidance for such decisions, but it also presupposes professional judgement capable of weighing needs, benefits and consequences in the specific situation.

When governance is characterised by goal congestion, this judgement risks being weakened, not because professionals lack willingness or competence, but because their scope for action is constrained. Rather than supporting holistic assessments, governance may contribute to decision-making being fragmented into separate requirements to be met individually. The ethical dilemma does not always become explicit, but is expressed instead in everyday situations where it becomes difficult to determine what is most appropriate here and now.

Within a person-centred logic, the jointly formulated goal of the intervention is central. From this perspective, goal congestion can be understood as a counterforce to precisely this shared goal orientation. When numerous overarching goals are given equal weight, the ability to let professional judgement guide action is undermined (Swedish Agency for Public Management, 2021).

## Keeping goals clear and the path professional

A recurring reflection concerns the importance of more clearly distinguishing between goals and implementation. When goals are understandable, relevant, and grounded in the realities of professional practice, conditions are created for engagement and accountability. When the path towards these goals is left open to professional judgement, space is also created for adaptation, innovation, and learning.

Monitoring remains necessary, but can advantageously be designed to support development rather than merely control. Ongoing dialogue, joint analysis, and feedback in close connection to practice can help maintain direction without unduly restricting room for action.

## Dialogue as a prerequisite for understanding

Disagreement between political decision-makers and professionals is not problematic in itself. On the contrary, differing perspectives can contribute to better decisions. For disagreement to become constructive, however, it requires respect for each other’s mandates and a willingness to understand the trade-offs made at different levels of the system.

Dialogue therefore needs to be more than a formal aspiration. It requires time, structure, and substance. Only then can it contribute to increased understanding of how goals are formulated, how priorities are set and what consequences they have in practice.

## Concluding reflection

When goal congestion arises, it is rarely an expression of a lack of engagement or ambition. Rather, it reflects a system that has gradually been expanded without sufficient consideration of the whole. In such a system, both policymakers and professionals risk losing their sense of direction, despite shared values and intentions (Swedish Agency for Public Management, 2023; Trust Delegation, 2018;).

Person-centredness can here offer a perspective that extends beyond the individual care encounter. As an approach, it emphasises the importance of clear goals, shared understanding, and trust in those responsible for implementation. When direction is clear and follow-up is shaped through dialogue rather than through a multitude of parallel demands, better conditions are created for professional responsibility (McCance and McCormack, 2025).

In this space, between political direction and professional everyday practice, the future of health and social care is shaped. It is here that the conditions for quality, sustainability, and trust are ultimately determined.

Policymakers need the courage to focus on fewer goals with greater clarity, rather than adding ever more requirements. Equally important is respecting the division of roles, where policymakers are responsible for defining what is to be achieved and professionals for determining how it is best carried out. Through this interplay, the conditions for sustainable and meaningful practice are created. When clarity guides decision-making, it becomes possible not only to achieve goals but also to develop operations in a way that feels both realistic and valuable to those working within them.
